# Visit of the Georgia Surgeon’s Club to New Orleans

**Published:** 1914-03

**Authors:** O. H. Matthews

**Affiliations:** Atlanta, Ga.


### VISIT OF THE GEORGIA SURGEONS' CLUB TO NEW ORLEANS.

By O. H. Matthews M. D., Atlanta, Ga.

The Georgia Surgeons' Club having been invited to hold its Convention in New Orleans this year, on the morning of February 27th, about ten o'clock, we arrived in that City, accompanied by prominent members of the profession from Alabama and South Carolina. A Committee representing the Surgeons of New Orleans met us and escorted us to our headquarters, at the St. Charles Hotel.

Immediately upon our arrival at the Hotel the party was divided in two, one group going to the Touro Infirmary and one to the Presbyterian Hospital. At each of these hospitals many interesting operative cases were ready. We were cordially received by the Attending Surgeons at these Institutions and conducted at once to the Operating Rooms, where special effort was made to give the best possible view of the work to each of the visitors.

At the Touro Infirmary Dr. Matas and other members of the Staff entertained us, giving us an opportunity of witnessing some very fine operative work, done in a manner and with a dexterity which will prove of much benefit to those who were fortunate enough to be present.

Those who went to the Presbyterian Hospital had the same courtesy extended them by the Staff of that Institution, and were unanimous in expressing their appreciation of the skill of the men who made the demonstrations.

At the conclusion of the morning's work we were assembled in the dining rooms of the Touro, where an elaborate luncheon had been prepared, to which we were quite ready to do full justice and to prove to ourselves that the pride which New Orleans takes in its culinary skill is well founded.

Immediately after luncheon we were escorted to the Richardson Memorial by the Entertainment Committee, at which place we were entertained by some of the Professors in the Laboratories of this branch of Tulane University. The Anatomical Museum of Prof. Souchon showed many wonderful specimens and was highly praised by every one.

From the Laboratories the members returned to the Hotel and divided into small parties, visiting several of the famous New Orleans cafes for dinner.

After dinner we were taken in charge by the Orleans Parish Medical Society, which entertained us with several symposiums, the subjects at hand being handled in a brilliant and thorough manner by the authors.

On the morning of the second day of our stay in the Crescent City we began work at 8:30 at the Charity Hospital. Here we were kept busy passing from one Operating room to another, and were privileged to witness many instructive and interesting operations, each one performed by a master surgeon.

For luncheon we again divided into small parties, following which some of the members visited historic places of interest in the old part of the City.

At 3 p. m., we met at the Hutchinson Memorial, the Medical Department of Tulane University, where we were very instructively entertained.

Each and every member of the party expressed himself as being well pleased with the information gained and was enthusiastic in his praise of the Surgeons for the thoroughness and ability with which they performed their work.

After boarding the train on our homeward journey a meeting of the Club was called by the President, Dr. E. C. Davis, at which time the Secretary, Dr. Harbin, was instructed to write a letter expressing our appreciation of the courtesies extended us while in New Orleans.

The following is a schedule of the program arranged for our entertainment:

New Orleans, Feb. 27th, 1914.

PRESBYTERIAN HOSPITAL—Dr. Cocram, Hysterectomy, 9:30; Dr. Batchelor, Goiter, 10:00.

TOURO INFIRMARY—Dr. Parham, Hernia, 9:30; Dr. Lanford, Path. Spec., 10:30; Dr. Miller, (1) Plastic, (2) Lap., 11:30; Room 2—Dr. Kohlmann—Lap., Lap. (Double pyo-scalp) 9:30; Dr. Matas, Exhibition of cases: 3 Liver cases, 2 Kidney Ossified hematoma. Bullet in Sella Turcica. Gigantism of lower extremities, patient and x-Ray. Demonstration of Kempton's Blood transfusion. 8 cases Cervical ribs x-Ray.

RICHARDSON MEMORIAL—Prof. Souchon, Anat. spec., 3:00; Drs. Duval and Couret, Surg. Path., 3:30; Dr. Mann, Surg. Phys., 4:00.

ORLEANS PARISH MEDICAL SOCIETY—8:00 to 8:45, Symposium Asites; Drs. Lemann, Matas, and Miller. 8:45 to 9:30, Spleno-Megaly; Drs. Lanford, Parham, and Weis. 9:30 to 11:00, Arthritis; Drs. Dupuy, Elliott, Gessner, Granger, Hume, and McIlhenny.

CHARITY HOSPITAL—(Miles Amphitheatre).
8:30 Dr. Kohlmann, Lap.; 10:00 Dr. Clark, Fibroid; 8:30 Dr. Nelken, Stricture Urethra; 10:00 Dr. Gessner, Laminectomy; 8:30 Dr. Cole, Appendix; 10:00 Dr. Stafford, Absc. of Liver; 8:30 Dr. Danna, Appendix; 10:00 Dr. Matas, 2 Aneurysms and a number of cases of unusual surgical interest.

CHARITY HOSPITAL—(Delgado).
8:30 Dr. Cocram, Fibroid; 10:00 Dr. Miller, Fibroid; 8:30 Dr. Parham, Appendix, Gastric Ulcer; 8:30 Dr. Allen, Rectal (Local); Dr. Maes, Hernia (Local); 8:30 Dr. Oeschner Arthrodosis (Ankle); 10:00 Dr. Perkins, Thoracic Tumor.

WARD CLINIC—9:00 to 11:30, Drs. Hume and Logan, Syphilis; Orthopodic Clinics by Dr. E. D. Fenner, Dr. E. S. Hatch, Dr. J. F. Oechsner.

CHARITY HOSPITAL—(Miles Amphitheatre) 11:30 to 1:30; 11:30-40 Dr. Martin, Bone Plate; 11:40-50 Dr. Allen, Empyema; 11:50-12 Dr. Perkins, Incomplete thoracoplasty; 12:00-10 Dr. Granger, Renal Calculus; 12:10-20 Dr. Hume, Diagnosis of T. B. Kidneys; 12:20-30 Dr. Miller, Hanley's Relief of oedema of arm; 12:30-40 Dr. Gessner; 12:40-50 Dr. Fenner, 3 cases; 12:50-1 Dr. Oeschner, Bone Grafts; 1:00-10 Dr. Wade, Specimen (Gastric-ulcer); 1:10-20 Dr. Danna, Imperforate Anus.

HUTCHINSON MEMORIAL—3:00 p. m. Dr. Bass, Malarial Plasmodia; 3:30 Dr. Allen, Demonstration of Intra Thoracic Surgery; 4:00 Dr. Maes, Demonstration of Artificial Peumothorax, and Hertles Method of injection of Gasserian Ganglion.

